# Contributors to Wisconsin’s persistent black-white gap in life expectancy

**DOI:** 10.1186/s12889-019-7145-y

**Published:** 2019-07-05

**Authors:** Max T. Roberts, Eric N. Reither, Sojung Lim

**Affiliations:** Utah State University, 0730 Old Main Hill, Logan, UT 84322 USA

**Keywords:** Life expectancy, Longevity, Cause of death, Racial disparities, Life table analysis, Wisconsin

## Abstract

**Background:**

Although the black-white gap in life expectancy has narrowed in the U.S., there is considerable variability across states. In Wisconsin, the black-white gap exceeds 6 years, well above the national average. Reducing this disparity is an urgent public health priority, but there is limited understanding of what contributes to Wisconsin’s racial gap in longevity. Our investigation identifies causes of death that contribute most to Wisconsin’s black-white gap in life expectancy among males and females, and highlights specific ages where each cause of death contributes most to the gap.

**Methods:**

Our study employs 1999–2016 restricted-use mortality data provided by the National Center for Health Statistics. After generating race- and sex-specific life tables for each 3-year period of observation (e.g., 1999–2001), we trace recent trends in the black-white life expectancy gap in Wisconsin. We subsequently conduct a series of analyses to decompose the black-white gap in three time periods into 13 separate causes and 19 different age groups.

**Results:**

In 2014–16, Wisconsin’s black-white gap in life expectancy was 7.34 years for males (67% larger than the national gap), and 5.61 years for females (115% larger than the national gap). Among males, homicide was the single largest contributor, accounting for 1.56 years of the total gap. Heart disease and cancer followed, contributing 1.43 and 1.42 years, respectively. Among females, heart disease and cancer were the two leading contributors to the gap, accounting for 1.12 and 1.00 years, respectively. Whereas homicide contributed most to the racial gap in male longevity during late adolescence and early adulthood, heart disease and cancer exerted most of their influence between ages 50–70 for both males and females. Other notable contributors were unintentional injuries (males), diabetes and cerebrovascular disease (females), and perinatal conditions (males and females).

**Conclusions:**

Our study identifies targets for future policy interventions that could substantially reduce Wisconsin’s racial gap in life expectancy. Concerted efforts to eliminate racial disparities in perinatal mortality and homicide early in the life course, and chronic conditions such as cancer and heart disease in later life, promise to help Wisconsin achieve the public health objective of racial parity in longevity.

**Electronic supplementary material:**

The online version of this article (10.1186/s12889-019-7145-y) contains supplementary material, which is available to authorized users.

## Background

In the United States, the black-white gap in life expectancy has been shrinking for decades—from 7.60 years in 1970, to 5.70 years in 2000, down to 3.60 years in 2013 [1–4]. Despite this encouraging trend, there remains substantial variability across states [2, 3, 5]. For example, Harper et al. [3] found black-white disparities of 3 years or less among non-Hispanics in Western states like Nevada, New Mexico, and Oregon. By contrast, the black-white life expectancy gap stagnated at a relatively high level in several Midwestern states between 1990 and 2009. In the case of Wisconsin, the gap actually increased over this time period, reaching 6.70 years among non-Hispanic females and 8.20 years among non-Hispanic males. As of 2009, this figure of 8.20 years was the widest black-white life expectancy gap for males or females in any of the 50 states [3]. Wisconsin may be emblematic of racial disparities in life expectancy found in other Midwestern states but, even among this select group, it is unusual in terms of the direction of trends (i.e., increasing black-white disparities) and the magnitude of the gap.

With approximately 6 million residents, Wisconsin is comparable to the state of Minnesota and also to European nations like Denmark and Finland in terms of total population size [6]. Approximately 7% of Wisconsin’s population is black [6], and the number of black residents in Wisconsin has increased in recent years [7]. The persistently large black-white disparity in longevity is therefore a major public health concern. To illustrate this point, consider the potential years of life lost among a single cohort of black infants in Wisconsin. In 2015, black females in Wisconsin gave birth to 6563 infants [8]. Assuming that infants in Wisconsin experience the series of age-specific death rates that Harper et al. [3] recently used to calculate black-white disparities in life expectancy (6.70 years among females and 8.20 years among males), this single cohort of 6563 black infants will lose nearly *fifty thousand* years of potential life, in total, due to the black disadvantage in longevity.

While this illustration highlights the public health impact of Wisconsin’s racial disparities in longevity, it is presently not clear what is contributing to those disparities. In an initial exploration of this issue, one recent investigation [5] assessed how five broadly-defined causes of death (cardiovascular disease, cancer, non-communicable disease, communicable disease, and injury) have perpetuated black-white gaps in life expectancy across U.S. states. This study found that black-white disparities in Midwestern states, including Wisconsin, were most affected by cardiovascular disease among both males and females [5]. In addition, cancer was a major contributor to Wisconsin’s black-white gap among males and females in 2013, which was the most recent period of observation in the study.

We build on these initial findings by examining Wisconsin in more detail through the following research aims: The first aim of our investigation is to trace the evolution of black-white longevity disparities in Wisconsin relative to the U.S. as a whole, showing how Wisconsin has diverged from national trends since the year 2000. Our second aim is to determine how 13 different causes of death (e.g., perinatal conditions and homicide) have contributed to Wisconsin’s black-white gap in life expectancy over time. Our third aim is to identify stages in the life course where different causes of death make the greatest contribution to the black-white gap. By expanding the range of potential contributors to black-white disparities in longevity and identifying specific ages where those causes of death contribute most to the gap, our study promotes targeted public health policies that are needed to reduce Wisconsin’s large black-white gap in life expectancy.

## Methods

### Data

To address our study aims, we employed data on death counts (*D*_*ij*_) and population estimates (*N*_*ij*_) to calculate death rates (*M*_*ij*_ = *D*_*ij*_/*N*_*ij*_) for selected subgroups (*i*) and periods of observation (*j*) in the state of Wisconsin. After securing permission from the National Center for Health Statistics (NCHS) and the Institutional Review Board at Utah State University (see *Ethics approval and consent to participate* at the end of this manuscript for details), we obtained multiple cause of death–all county micro data files for 1999–2016 from the NCHS [9]. These data provide complete counts of total and specific causes of death by period of observation, sex, age, ethnicity and race. For denominators, we used U.S. Census population estimates with bridged race categories [10], consistent with prior research in this area [3]. These population estimates cover the same time periods and demographic characteristics as the numerators.

### Measures

We restricted our analyses to non-Hispanics in Wisconsin, as Hispanics are distinct from non-Hispanic blacks and whites in terms of social and economic factors and subsequent health outcomes [2, 11]. In addition, we examined males and females separately as the contributors to the black-white gap in life expectancy vary by sex [5]. Using the measures of race, ethnicity and sex provided by the NCHS, we focused on the following four race-sex groups in this study: non-Hispanic black males; non-Hispanic black females; non-Hispanic white males; and non-Hispanic white females (hereafter black males, black females, white males, and white females). For each race-sex group, we conducted life table analyses to identify specific life stages where black-white disparities are most pronounced. In these life tables, we categorized age into (1) less than 1 year of age, (2) one to 4 years of age, (3) five-year age groups ranging from 5–9 to 80–84, and (4) an open-ended category for ages 85 and older [12].

Causes of death were categorized in accordance with the International Classification of Diseases (ICD), 10th revision [13]. We focused on 13 causes of death that are either leading causes of death nationwide [14] or suspected contributors to the black-white mortality gap [4]. These causes of death include accidents (unintentional injuries), assault (homicide), cerebrovascular disease, diabetes mellitus, heart disease, human immunodeficiency virus (HIV), hypertension, influenza/pneumonia, intentional self-harm (suicide), liver disease, malignant neoplasms (cancer), perinatal conditions, respiratory disease, and a residual category for all other causes of death.

### Analyses

We used Microsoft Excel [15] in our analyses, which aggregated 3 years of death (*D*_*ij*_) and population (*N*_*ij*_) data into individual cross-sections of time to minimize random fluctuations in death rates (*M*_*ij*_) for each age-race-sex subgroup. To address study aim 1 (tracing black-white disparities since 2000), we converted age-race-sex-specific death rates into probability estimates [12] and generated period life tables for each group (i.e., black and white males and females) in Wisconsin as well as the entire U.S. In the process of converting rates to probabilities, we employed graduation techniques to estimate the average person-years lived among persons who died between the ages of *x* to *x + n* (_n_*a*_x_).^1^ We then arrayed life expectancies (*e*_*0*_) derived from these life tables into three-year moving averages, spanning the time period 2000 (i.e., 1999–2001) to 2015 (i.e., 2014–2016). These analyses facilitated visualization of trends in Wisconsin’s black-white life expectancy gap, relative to the rest of the nation.

To address aim 2 (identifying causes of death that contributed most to black-white disparities), we decomposed the overall black-white *e*_*0*_ gap into portions attributable to 13 causes of death, as well as a residual category for all other causes of death. We employed the age and cause decomposition method with discrete data following prior work [5, 16, 17]. For aim 3 (identifying life stages where specific causes of death had the greatest impact), we calculated the number of years that each cause of death contributed to the overall black-white *e*_*0*_ gap for each age group. We assessed aims 2–3 at three different time points, spanning our entire period of observation: 2000 (1999–2001), 2008 (2007–2009), and 2015 (2014–2016).

## Results

**Fig. 1:**
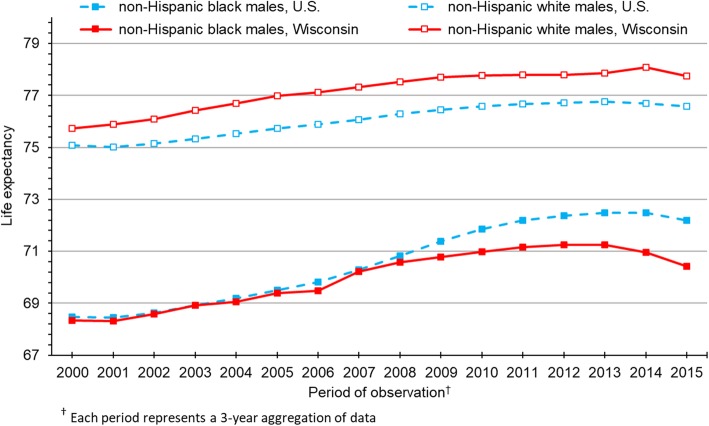
Life expectancy for non-Hispanic black and non-Hispanic white males in Wisconsin and the U.S.

Figure 1 shows life expectancy trends from 2000 to 2015 for black and white males in Wisconsin and the U.S. as a whole. At the beginning of this study period, black-white gaps in life expectancy were similar for males in Wisconsin (7.40 years) and the U.S. (6.60 years). Among U.S. males, the black-white gap improved over the next 15 years, narrowing to 4.39 years in 2015. Conversely, the racial gap among males persisted in Wisconsin throughout the period, showing no consistent indications of improvement. In 2015, black males in Wisconsin had a life expectancy of 70.41 years, compared to 77.75 years for white males. This gap of 7.34 years was 67% larger than the black-white gap among U.S. males (4.39 years).

**Fig. 2:**
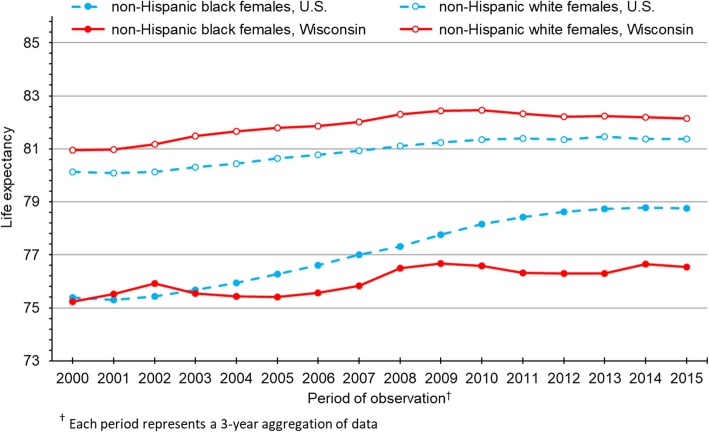
Life expectancy for non-Hispanic black and non-Hispanic white females in Wisconsin and the U.S.

Figure 2 presents life expectancy trends for black and white females in Wisconsin, again including the U.S. as a basis for comparison. As we observed among males, the black-white gap narrowed considerably between 2000 and 2015 among U.S. females, reaching 2.61 years in 2015. By contrast, the black-white gap in life expectancy remained wide among females in Wisconsin, with relatively minor fluctuations across the period. In 2015, black and white females in Wisconsin could expect to live 76.54 and 82.15 years, respectively, creating a gap of 5.61 years—115% larger than the black-white gap among U.S. females.

Whereas U.S. black males and females enjoyed steady gains in life expectancy for most of the study period, notable improvements for black Wisconsinites ended around 2008 for both males (Fig. 1) and females (Fig. 2). Consequently, the divergence in life expectancy trends between U.S. and Wisconsin blacks was pronounced after 2008, for males and females alike. As life expectancy among U.S. blacks outpaced Wisconsin blacks, U.S. blacks narrowed the gap with U.S. whites, who experienced relatively slow gains in life expectancy over the study period. Despite those gains, life expectancy among U.S. whites consistently trailed Wisconsin whites by about a year, regardless of sex.

In Table 1, we show how 13 causes of death contributed to Wisconsin’s black-white life expectancy gap for males in 2000, 2008 and 2015. Our decomposition analyses revealed that homicide, heart disease and malignant neoplasms (cancer) were consistently the largest contributors during this study period. In 2015, these three causes of death were responsible for 60% of the longevity difference between black and white males. Homicide was the greatest contributor in 2015, accounting for 1.56 years of the 7.34-year gap, followed by heart disease and cancer (1.43 and 1.42 years, respectively). Between 2000 and 2015, the contribution of cerebrovascular disease, HIV and suicide all declined by at least 0.2 years. These improvements were offset by heart disease and unintentional injuries, which grew in their contribution to the longevity gap by 0.43 and 0.52 years, respectively. Due to these changes, unintentional injuries displaced cerebrovascular disease in 2015 as a top-five contributor. Perinatal conditions were consistently a top-five contributor to longevity disparities among males, accounting for approximately 5–6% of the gap at each time point.

**Table 1:**
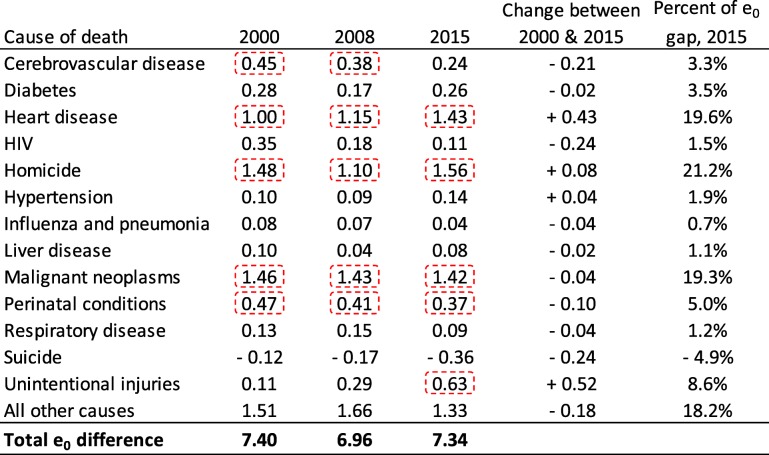
Contribution of 13 causes of death, in years, to life expectancy (e_0_) differences between non-Hispanic black and non-Hispanic white males in Wisconsin

Note: Each period represents a 3-year average (e.g., 1999–2001). Values highlighted by dotted rectangles indicate the top five contributors in each study period

Table 2 decomposes the non-Hispanic black-white gap among females into 13 causes of death for each study period. Heart disease and cancer were the two most important contributors that, in combination, accounted for 36% of the gap in 2000, 42% of the gap in 2008 and 38% of the gap in 2015. Other top-five contributors at each time point were perinatal conditions, cerebrovascular disease, and diabetes. In 2015, each of these conditions accounted for approximately 5 months of the 5.61-year gap. Altogether, the leading five contributors were responsible for 59% of the black-white gap in life expectancy among females in 2015. Whereas homicide was the leading contributor to the gap among males in 2000 and 2015, it was consistently the sixth largest contributor among females, accounting for 0.24 years in 2015—a relatively small but certainly not trivial amount. Between 2000 and 2015, diabetes and heart disease declined in their contribution to the gap by 0.17 and 0.20 years, respectively. Unintentional injuries and especially cancer offset those improvements, as their contribution to the gap increased by 0.15 and 0.26 years, respectively.

**Table 2:**
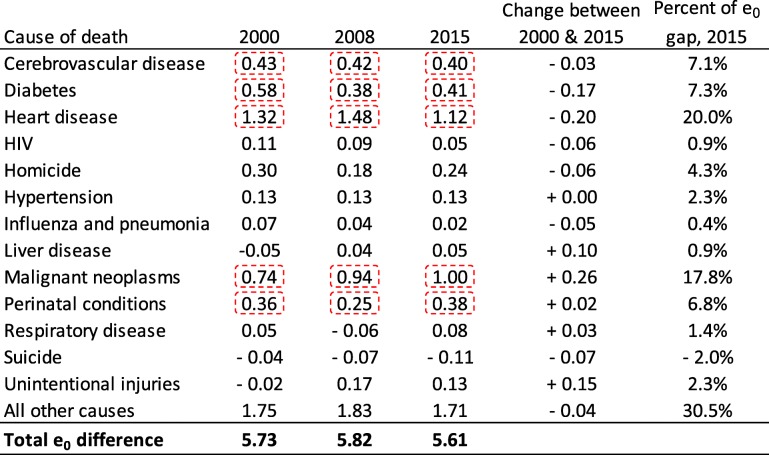
Contribution of 13 causes of death, in years, to life expectancy (e_0_) differences between non-Hispanic black and non-Hispanic white females in Wisconsin

Note: Each period represents a 3-year average (e.g., 1999–2001). Values highlighted by dotted rectangles indicate the top five contributors in each study period

**Fig. 3:**
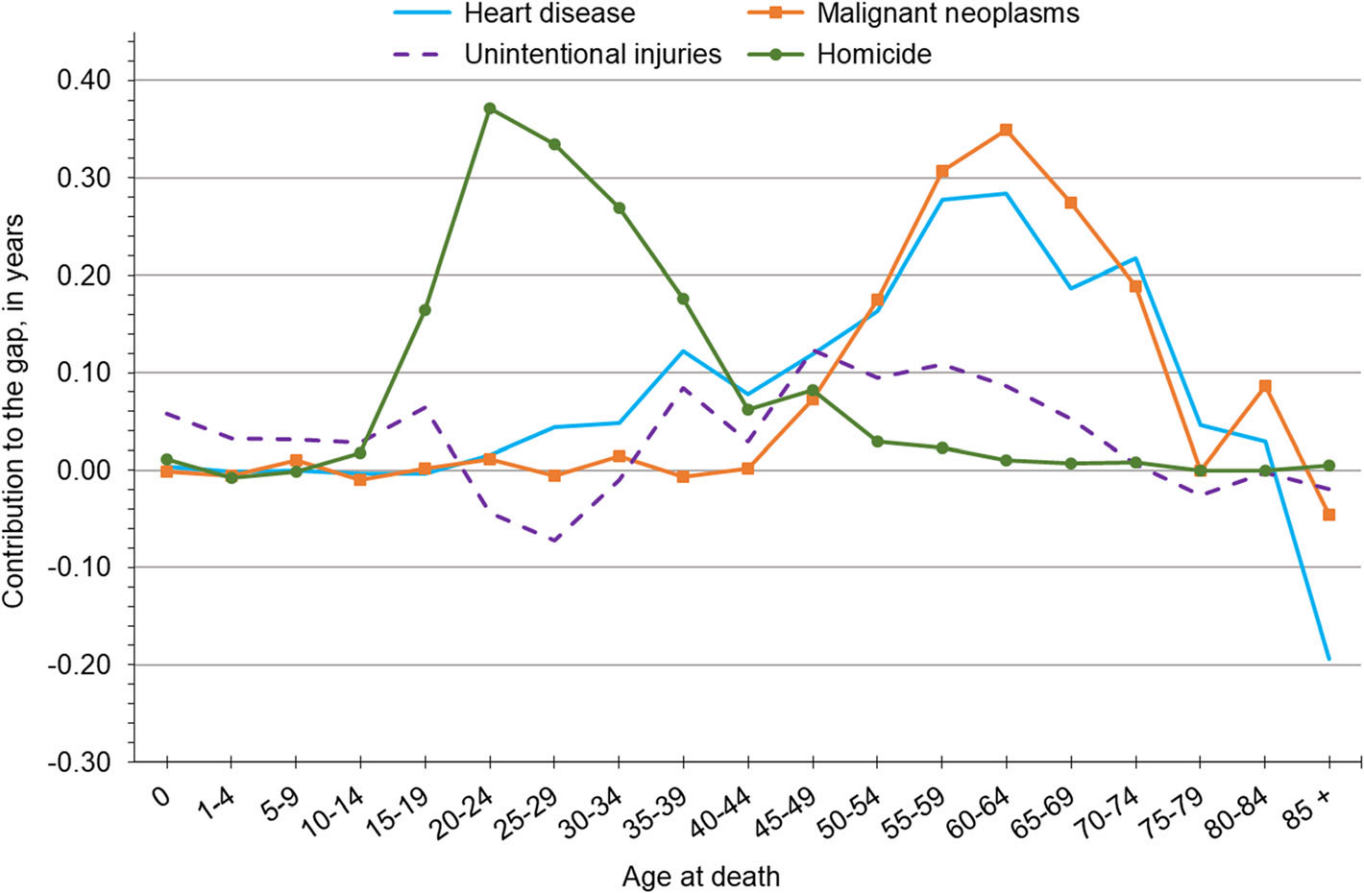
Decomposition of Wisconsin’s black-white gap in life expectancy by age and cause of death for non-Hispanic males, 2014–16

In Fig. 3, we present results from decomposition analyses for Wisconsin males in 2015 to illustrate how much each major cause of death contributed to the black-white gap in life expectancy across various life stages. To ease interpretation of this figure, we only included the four largest contributing causes of death and excluded perinatal conditions (the fifth leading contributor), since its impact is concentrated in the first age group. We also focus on results for 2015, as the age patterns we observed were broadly consistent over time; for complete age and cause decomposition results in 2000, 2008 and 2015, see Additional files 1, 2 and 3. The sharp spike in homicide indicates that it contributed most to the black-white gap in life expectancy among males between ages 15–39. Within this age range, the largest contributions occurred at ages 20–24 (0.37 years) and 25–29 (0.33 years). Heart disease and cancer played a major role in the black-white gap later in life, specifically between ages 50–74. Within that 25-year span, heart disease contributed most to the gap at ages 55–59 and 60–64 (0.28 years for each age group). Cancer’s impact was also most pronounced at ages 60–64 (0.35 years) and, overall, it followed much the same age pattern as heart disease. Unintentional injuries appeared to be less age dependent than other causes of death among males, as their contribution fluctuated across the life course.

**Fig. 4:**
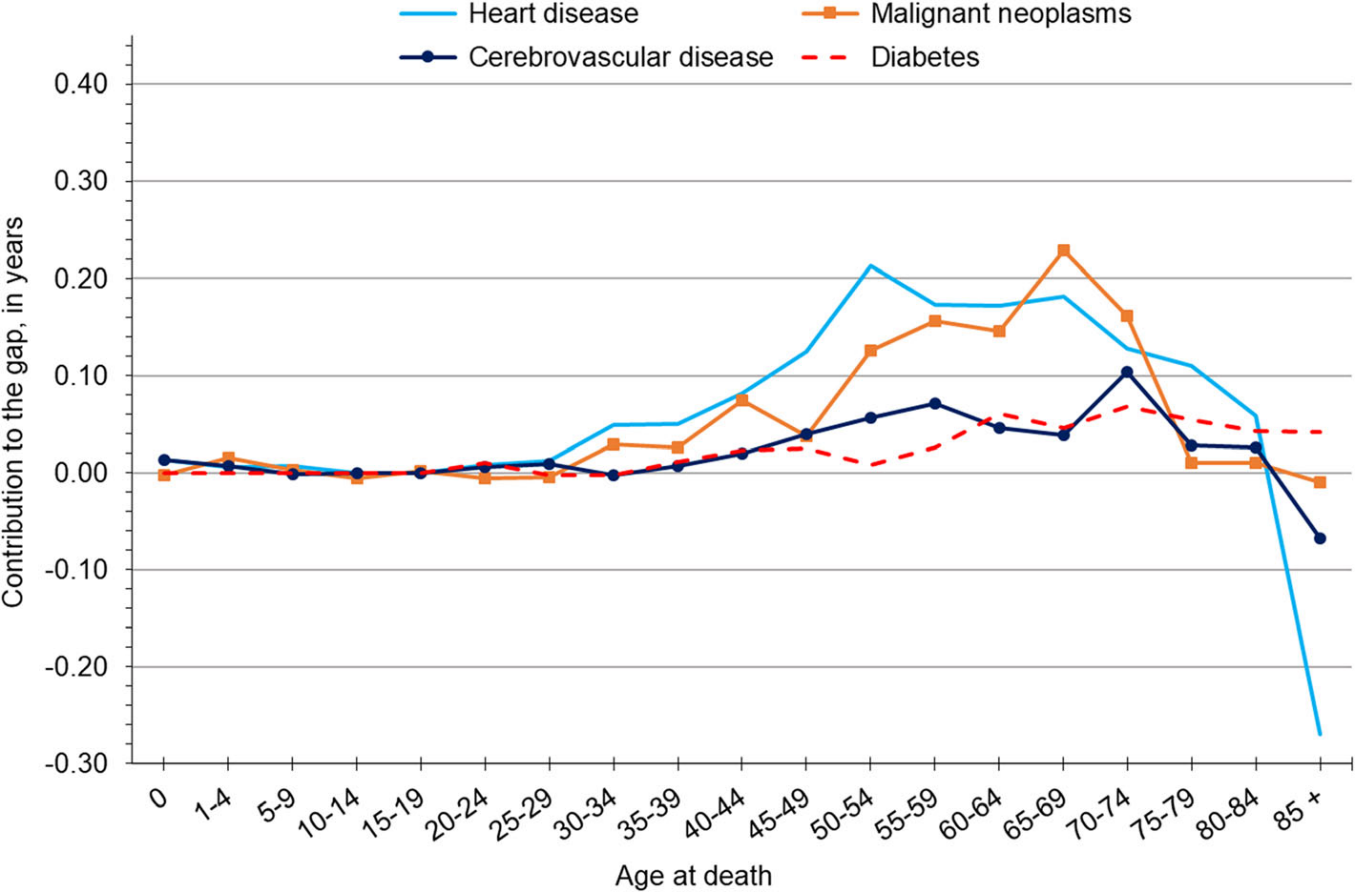
Decomposition of Wisconsin’s black-white gap in life expectancy by age and cause of death for non-Hispanic females, 2014–16

We highlight findings from age and cause decomposition analyses of the black-white life expectancy gap in 2015 for Wisconsin females in Fig. 4. As before, we included the four leading contributors and concentrated on the most recent findings, as age patterns were generally stable across periods; interested readers may consult Additional files 4, 5, and 6 for complete age and cause decomposition tables for females in all three periods. Among females in 2015, heart disease contributed most to the black-white gap between ages 50–69. Within that 20-year range, heart disease contributed most among women aged 50–54, accounting for 0.21 years of the black-white gap. The steep drop-off for heart disease after age 85 represented gains in life expectancy for black females—a finding that also held true for black males. Cancer contributed most to the black-white gap for females between ages 65–74, accounting for 0.39 years during this stage of the life course. Cerebrovascular disease was a major contributor for women aged 70–74, accounting for 0.10 years of the gap. The contribution of diabetes increased gradually after age 50, peaking at 0.07 years at age 70–74.

As shown in Figs. 1 and 2, life expectancy in the year 2000 was essentially identical for blacks in the U.S. and Wisconsin. However, black Wisconsinites failed to keep pace with their U.S. counterparts in subsequent years. The divergence between U.S. and Wisconsin blacks is especially notable after 2008. To help understand this phenomenon, we conducted supplemental analyses to decompose life expectancy gaps between U.S. and Wisconsin blacks in 2008 and 2015 (Additional files 7, 8, 9 and 10). For males and females in both periods of observation, malignant neoplasms contributed most to these gaps, followed by unintentional injuries. Among males, homicide also played an increasingly important role, contributing essentially nothing to the gap in 2008 but 0.43 years in 2015. In fact, just three causes of death in 2015—cancer, unintentional injuries and homicide—accounted for 83% of the 2.03-year gap in life expectancy between black males in the U.S. and Wisconsin. Among females, respiratory disease, perinatal conditions and cerebrovascular disease followed cancer and unintentional injuries as the largest contributors the gap in 2015. In combination, these five conditions accounted for 54% of the 2.44-year gap in life expectancy between U.S. and Wisconsin black females. Furthermore, in absolute terms, each of these five conditions contributed more to the gap in 2015 than it did in 2008.

## Discussion

Using recent and complete mortality data, our investigation affirms the persistence of large black-white longevity disparities in Wisconsin. In the latest 3-year period of observation (2014–16), black males and females could expect to live 7.34 and 5.61 years less, respectively, than their white counterparts. To effectively address those racial disparities, it is important to identify major contributors to the gap. One prior investigation [5] found that, as of 2013, cardiovascular disease and cancer were the two leading contributors to the black-white life expectancy gap in Wisconsin for both males and females. While our study affirmed the continuing importance of those conditions, it also found that homicide was the single greatest contributor to the black-white gap in life expectancy among males. The contribution of homicide was strongly concentrated in late adolescence and early adulthood, reflecting high rates of homicide among young black males in Wisconsin [18, 19]. It also reflects Wisconsin’s generally high rate of homicide among blacks. In 2015, the rate of black homicide victimization in Wisconsin was 37 deaths per 100,000 residents, which was second highest in the nation and twice the national average for blacks [20]. Indeed, even among females, homicide accounted for about 3 months of the black-white gap in life expectancy in 2015. We made these discoveries by disaggregating the broad “injury” category used by Riddell et al. [5] into its main constituent components—namely homicide, unintentional injuries and suicide—and by identifying stages of the life course where each cause of death contributed most to the gap. Reducing black-white disparities in longevity will require concerted efforts to reduce levels of violence among teenagers and young adults, which are unfortunately endemic in some of Wisconsin’s highly segregated black communities [20, 21].

Through our investigation, we made additional discoveries by separating the cardiovascular and non-communicable disease categories in Riddell et al. [5] into heart disease, cerebrovascular disease, diabetes and hypertension. Heart disease was consistently the largest contributor to the black-white gap in life expectancy among females, and it made an even larger absolute contribution to the gap among males (1.43 years) in 2015. Conversely, cerebrovascular disease and diabetes generally made larger contributions to the gap among females, both in relative and absolute terms. The large contribution of diabetes suggests that obesity may play a relatively important role in generating longevity disparities among females. Data from the Behavioral Risk Factor Surveillance System (BRFSS) [22] covering the period 2014–16 support that contention. Nearly 44% of black females in Wisconsin were obese in 2014–16, compared to 29% of white females. Although BRFSS data also showed disparities among males in Wisconsin, they were small by comparison, with 38% of black males and 32% of white males indicating obesity, respectively.

The absolute contribution of cancer to black-white differences in life expectancy was larger among males than females, despite comparable relative contributions. Large contributions from cancer and heart disease—but a relatively small contribution from diabetes—suggest that smoking may play an important role in generating black-white longevity disparities among males. Data from the 2014–16 BRFSS [22] support this interpretation; 34% of black males in Wisconsin were current smokers, compared to 17% of white males. By comparison, black-white smoking disparities were small among females in Wisconsin; 20% of black females and 14% of white females smoked in 2014–16. The strikingly similar pattern that cancer and heart disease followed over the life course of males may also implicate smoking as a key determinant of those causes of death. Among females, heart disease contributed most to the black-white gap earlier in life than cancer, which could point to different underlying causes. Although further research is needed to elucidate these associations, it seems clear that policies and programs designed to reduce high rates of smoking and obesity among black Wisconsinites would help reduce large racial disparities in life expectancy.

Whereas Riddell et al. [5] included perinatal conditions in a broad residual category of “all other” conditions, we examined this cause of death separately. This led us to discover that perinatal mortality is a perennial top-five contributor to the black-white gap in life expectancy for both males and females in Wisconsin. This finding is consistent with prior research ranking Wisconsin’s black infant mortality rate as the highest in the U.S [23]. In 2010, blacks accounted for 24% of infant deaths in Wisconsin, even though black infants comprised only 10% of all live births [24]. Although most Wisconsinites have access to health insurance, BRFSS data from 2014–16 [22] indicate that 54% of white women received insurance through their employers, compared to just 26% of black women. This may have implications with respect to the availability and quality of healthcare options for some black women in Wisconsin. Reducing perinatal mortality among blacks in Wisconsin will require programs and policies capable of improving maternal health conditions and providing universal access to high-quality prenatal care [25].

Underlying black-white disparities in mortality and related health behaviors such as smoking are substantial social and economic inequalities. Prior research has pointed to socioeconomic disadvantages as “fundamental causes” of suboptimal health among blacks because they limit access to resources, including health-promoting goods and services, and create chronic stressors that affect a wide range of health outcomes [26, 27]. Highlighting these disadvantages are statistics on employment, poverty and education. In 2017, the unemployment rate in Wisconsin was 9.3% among black adults but only 2.6% among white adults [28]. Among those employed in Wisconsin, approximately 23% of black men and 35% of black women earned less than $12 dollars per hour in 2017; corresponding figures for white men and women were 12% and 20% respectively [29]. Due to these differences in employment and wages, 36.2% of Wisconsin’s black residents lived in poverty, compared to 9.4% of white residents [30].

It is also worth noting that the Great Recession hit Wisconsin’s black residents particularly hard. The unemployment rate among blacks in 2005 was about 10%, both in Wisconsin and the U.S. [31]. By 2010, unemployment ballooned to about 25% among black Wisconsinites, compared to 15% among U.S. blacks and 8% among whites in Wisconsin and the U.S. While blacks in Wisconsin slowly recovered economically after 2010, they still trail U.S. blacks and especially whites in Wisconsin. As of 2015, Wisconsin ranked 2nd or 3rd worst among all U.S. states with respect to black-white disparities in unemployment, labor force participation, household income and poverty [32]. Although we lack the data to make causal inferences, we note that the extraordinarily difficult economic situation experienced by black Wisconsinites during and after the Great Recession corresponds to our finding that the longevity gap between blacks in the U.S. and Wisconsin widened substantially after 2008.

In addition to economic disparities, Wisconsin has substantial racial differences in educational opportunities and attainment. To illustrate, a national benchmark exam administered to eighth graders in 2015 found that, on average, black children scored 48 points lower than white children in Wisconsin—the largest gap in any of the 50 states [33]. Prior research has shown that racial differences in educational attainment are tied to longevity disparities [34], which may help explain the persistently large black-white life expectancy gap in Wisconsin. However, moving beyond plausible speculation will require additional research designed to elucidate causal associations between socioeconomic conditions in Wisconsin and racial disparities in longevity.

Our study has some notable strengths and limitations. One important strength is our use of restricted-use data from the NCHS [9]. Whereas previous studies on black-white longevity differences in U.S. states [3, 5] addressed missing data in CDC WONDER [35] through sophisticated imputation procedures, we eliminated the need for statistical estimates by accessing restricted-use data. Another key strength is the focus on 13 specific causes of death, which facilitated a number of important new discoveries. Additionally, our study showed how each major cause of death contributed to the black-white gap in life expectancy across the life course of males and females, providing policymakers with information not previously available. Although these 13 causes of death accounted for 82% and 70% of the 2015 black-white life expectancy gap among males and females, respectively, a limitation of our study is its inability to account fully for these gaps. Future research could consider what additional causes may account for the “all other” residual categories, and what this may imply with respect to the underlying determinants of black-white disparities. Another limitation of our investigation is that socioeconomic data are not available to pair with the NCHS mortality files. Consequently, although we note substantial social and economic disadvantages among blacks in Wisconsin, we are not able to quantify how much they contribute to black-white longevity disparities. As noted, future research would benefit from exploring socioeconomic disadvantages to determine the extent to which they affect major contributors to Wisconsin’s black-white gap in life expectancy.

Future research could also explore other racial and ethnic longevity disparities in Wisconsin. According to a recent report from the Wisconsin Department of Health Services [36], life expectancy in 2010–14 among Hispanics (86.9 years) and Asians (85.3 years) exceeded whites (79.8 years), blacks (73.8 years) and American Indians (72.8 years). These racial and ethnic gaps in longevity pertain to both males and females. The considerable life expectancy advantage among Hispanics is a paradox, as poverty is more prevalent in Wisconsin among Hispanics than it is among whites [37, 38]. Previous research has pointed to migration dynamics and relatively low rates of smoking among Hispanics as potential explanations for this paradox in national data [39]. Although these issues are beyond the scope of our current investigation, they provide opportunities for further research on Wisconsin’s racial and ethnic gaps in life expectancy.

## Conclusions

Wisconsin’s persistent black-white gap in life expectancy is an urgent public health issue. Our investigation revealed that homicide in adolescence and young adulthood contributed most to the gap among males, followed by heart disease and cancer later in life. Among females, heart disease and cancer were the two leading contributors, with the peak contribution occurring earlier in life for heart disease than cancer. Perinatal conditions also made large contributions to the gap for males and females. Concerted efforts to eliminate racial disparities in perinatal mortality and homicide early in the life course, and chronic conditions such as cancer and heart disease in later life, promise to help Wisconsin achieve the public health objective of racial parity in longevity.

## Additional files

Additional file 1: Age and cause decomposition of the 7.40-year difference in life expectancy between non-Hispanic black and non-Hispanic white males in Wisconsin, 1999–01. This table shows the contribution, in years, of each age group and cause of death to the total difference in life expectancy between non-Hispanic black and non-Hispanic white males in Wisconsin from 1999–01. (PDF 93 kb)

Additional file 2: Age and cause decomposition of the 6.96-year difference in life expectancy between non-Hispanic black and non-Hispanic white males in Wisconsin, 2007–09. This table shows the contribution, in years, of each age group and cause of death to the total difference in life expectancy between non-Hispanic black and non-Hispanic white males in Wisconsin from 2007–09. (PDF 95 kb)

Additional file 3: Age and cause decomposition of the 7.34-year difference in life expectancy between non-Hispanic black and non-Hispanic white males in Wisconsin, 2014–16. This table shows the contribution, in years, of each age group and cause of death to the total difference in life expectancy between non-Hispanic black and non-Hispanic white males in Wisconsin from 2014–16. (PDF 95 kb)

Additional file 4: Age and cause decomposition of the 5.73-year difference in life expectancy between non-Hispanic black and non-Hispanic white females in Wisconsin, 1999–01. This table shows the contribution, in years, of each age group and cause of death to the total difference in life expectancy between non-Hispanic black and non-Hispanic white females in Wisconsin from 1999–01. (PDF 93 kb)

Additional file 5: Age and cause decomposition of the 5.82-year difference in life expectancy between non-Hispanic black and non-Hispanic white females in Wisconsin, 2007–09. This table shows the contribution, in years, of each age group and cause of death to the total difference in life expectancy between non-Hispanic black and non-Hispanic white females in Wisconsin from 2007–09. (PDF 95 kb)

Additional file 6: Age and cause decomposition of the 5.61-year difference in life expectancy between non-Hispanic black and non-Hispanic white females in Wisconsin, 2014–16. This table shows the contribution, in years, of each age group and cause of death to the total difference in life expectancy between non-Hispanic black and non-Hispanic white females in Wisconsin from 2014–16. (PDF 93 kb)

Additional file 7: Age and cause decomposition of the 0.24-year difference in life expectancy between non-Hispanic black males in Wisconsin and non-Hispanic black males in the U.S., 2007–09. This table shows the contribution, in years, of each age group and cause of death to the total difference in life expectancy between non-Hispanic black males in Wisconsin and non-Hispanic black males in the U.S. from 2007–09. (PDF 94 kb)

Additional file 8: Age and cause decomposition of the 2.03-year difference in life expectancy between non-Hispanic black males in Wisconsin and non-Hispanic black males in the U.S., 2014–16. This table shows the contribution, in years, of each age group and cause of death to the total difference in life expectancy between non-Hispanic black males in Wisconsin and non-Hispanic black males in the U.S. from 2014–16. (PDF 94 kb)

Additional file 9: Age and cause decomposition of the 0.92-year difference in life expectancy between non-Hispanic black females in Wisconsin and non-Hispanic black females in the U.S., 2007–09. This table shows the contribution, in years, of each age group and cause of death to the total difference in life expectancy between non-Hispanic black females in Wisconsin and non-Hispanic black females in the U.S. from 2007–09. (PDF 94 kb)

Additional file 10: Age and cause decomposition of the 2.44-year difference in life expectancy between non-Hispanic black females in Wisconsin and non-Hispanic black females in the U.S., 2014–16. This table shows the contribution, in years, of each age group and cause of death to the total difference in life expectancy between non-Hispanic black females in Wisconsin and non-Hispanic black females in the U.S. from 2014–16. (PDF 95 kb)

## Data Availability

Data supporting the findings in this study are available from the National Center for Health Statistics. Restrictions apply to the availability of these data, which were used under license for the current study.

## Abbreviations

ICD: International Classification of Diseases
NCHS: National Center for Health Statistics
U.S.: United States

## Footnotes

1 We convert age-specific death rates to probabilities between ages *x* and *x* + *n* using _*n*_*a*_*x*_ as follows: _*n*_*q*_*x*_ = (*n* · _*n*_*m*_*x*_) / 1 + (*n* – _*n*_*a*_*x*_) · _*n*_*m*_*x*_, where _*n*_*q*_*x*_ is the probability of dying between ages *x* and *x* + *n*, *n* is the number of observed years within the age interval, _*n*_*m*_*x*_ is the age-specific death rate between ages *x* and *x* + *n*, and _*n*_*a*_*x*_ is the average person-years lived between ages *x* and *x* + *n* for persons dying in the age interval. For more information, please refer to *Demography: Measuring and Modeling Population Processes* by S.H. Preston et al. [12].
