# Supplementary material for: Contributors to Wisconsin’s persistent black-white gap in life expectancy
Source: BMC Public Health. 2019 Jul 5;19:891. doi: 10.1186/s12889-019-7145-y (PMC6612087; doi:10.1186/s12889-019-7145-y)
Supplement: Supplementary file 1 — Age and cause decomposition of the 7.40-year difference in life expectancy between non-Hispanic black and non-Hispanic white males in Wisconsin, 1999–01. This table shows the contribution, in years, of each age group and cause of death to the total difference in life expectancy between non-Hispanic black and non-Hispanic white males in Wisconsin from 1999–01. (PDF 93 kb) [file 12889_2019_7145_MOESM1_ESM.pdf]

Additional file 1. Age and cause decomposition of the 7.40-year difference in life expectancy between non-Hispanic black and non-Hispanic white males in Wisconsin, 1999-01

Age groups	Cerebrovascular disease	Diabetes	Heart disease	HIV	Homicide	Hypertension	Influenza & pneumonia	Liver disease	Malignant neoplasms	Perinatal conditions	Respiratory disease	Suicide	Unintentional injuries	All other causes	Total
0	0.00	0.00	0.00	0.00	0.02	0.00	0.02	0.00	0.00	0.47	0.01	0.00	0.03	0.33	0.89
1-4	0.00	0.00	0.01	0.00	0.02	0.00	0.00	0.00	0.00	0.00	0.00	0.00	0.03	0.01	0.07
5-9	0.00	0.00	0.00	0.00	0.01	0.00	0.00	0.00	-0.01	0.00	0.02	0.00	0.03	0.00	0.04
10-14	0.00	0.00	0.00	0.00	0.02	0.00	0.00	0.00	0.01	0.00	0.01	0.00	0.03	0.03	0.09
15-19	0.00	0.01	0.01	0.00	0.33	0.00	0.00	0.00	0.01	0.00	0.01	0.00	-0.08	0.00	0.29
20-24	0.00	0.00	0.01	0.00	0.44	0.00	0.00	0.00	0.01	0.00	0.00	-0.01	-0.10	0.11	0.44
25-29	0.00	0.00	0.02	0.03	0.21	0.00	0.00	0.01	0.00	0.00	0.00	-0.03	-0.04	0.03	0.22
30-34	0.00	0.00	0.02	0.03	0.14	0.01	0.00	0.00	0.02	0.00	0.01	-0.01	0.00	0.08	0.29
35-39	0.01	0.01	0.08	0.09	0.14	0.00	0.00	0.01	0.02	0.00	0.01	-0.03	0.04	0.07	0.44
40-44	0.04	0.01	0.03	0.10	0.07	0.00	0.01	0.01	0.12	0.00	0.01	-0.01	0.05	0.12	0.56
45-49	0.06	0.03	0.12	0.03	0.03	0.01	0.01	0.04	0.10	0.00	0.01	0.00	0.05	0.13	0.62
50-54	0.04	0.04	0.24	0.03	0.02	0.00	0.01	0.04	0.15	0.00	0.01	-0.01	0.08	0.15	0.81
55-59	0.07	0.05	0.22	0.03	0.01	0.01	0.00	0.01	0.24	0.00	0.01	-0.01	0.03	0.14	0.83
60-64	0.05	0.03	0.19	0.00	0.00	0.01	0.01	-0.02	0.24	0.00	0.01	0.00	0.00	0.11	0.62
65-69	0.09	0.06	0.14	0.00	0.00	0.01	0.00	0.01	0.22	0.00	0.06	0.00	0.01	0.10	0.69
70-74	0.04	0.01	0.04	0.00	0.01	0.01	0.00	-0.01	0.15	0.00	0.01	0.00	0.00	0.10	0.36
75-79	0.04	0.02	0.00	0.00	0.00	0.01	0.00	0.00	0.12	0.00	-0.01	0.00	-0.01	0.02	0.18
80-84	0.03	0.01	0.03	0.00	0.00	0.01	0.00	0.00	0.08	0.00	-0.02	0.00	0.00	0.05	0.19
85 +	-0.01	0.01	-0.15	0.00	0.00	0.02	0.01	0.00	-0.01	0.00	0.01	0.00	-0.02	-0.08	-0.24
**Totals**	**0.45**	**0.28**	**1.00**	**0.35**	**1.48**	**0.10**	**0.08**	**0.10**	**1.46**	**0.47**	**0.13**	**-0.12**	**0.11**	**1.51**	**7.40**

Note: Table entries represent the contribution, in years, to the total difference in life expectancy. Some of these values may be negative.
