# Supplementary material for: Contributors to Wisconsin’s persistent black-white gap in life expectancy
Source: BMC Public Health. 2019 Jul 5;19:891. doi: 10.1186/s12889-019-7145-y (PMC6612087; doi:10.1186/s12889-019-7145-y)
Supplement: Supplementary file 2 — Age and cause decomposition of the 6.96-year difference in life expectancy between non-Hispanic black and non-Hispanic white males in Wisconsin, 2007–09. This table shows the contribution, in years, of each age group and cause of death to the total difference in life expectancy between non-Hispanic black and non-Hispanic white males in Wisconsin from 2007–09. (PDF 95 kb) [file 12889_2019_7145_MOESM2_ESM.pdf]

**Additional file 2.** Age and cause decomposition of the 6.96-year difference in life expectancy between non-Hispanic black and non-Hispanic white males in Wisconsin, 2007-09

| Age groups | Cerebrovascular disease | Diabetes | Heart disease | HIV | Homicide | Hypertension | Influenza & pneumonia | Liver disease | Malignant neoplasms | Perinatal conditions | Respiratory disease | Suicide | Unintentional injuries | All other causes | Total |
|---|---|---|---|---|---|---|---|---|---|---|---|---|---|---|---|
| 0 | 0.00 | 0.00 | 0.02 | 0.00 | 0.05 | 0.00 | 0.01 | 0.00 | 0.01 | 0.39 | 0.00 | 0.00 | 0.06 | 0.17 | 0.70 |
| 1-4 | 0.00 | 0.00 | 0.01 | 0.00 | 0.02 | 0.00 | 0.00 | 0.00 | 0.00 | 0.01 | 0.00 | 0.00 | 0.01 | 0.00 | 0.05 |
| 5-9 | 0.00 | 0.00 | 0.00 | 0.00 | 0.00 | 0.00 | 0.00 | 0.00 | -0.01 | 0.01 | 0.01 | 0.00 | 0.00 | 0.01 | 0.02 |
| 10-14 | 0.00 | 0.00 | 0.00 | 0.00 | 0.00 | 0.00 | 0.00 | 0.00 | 0.00 | 0.00 | 0.01 | 0.01 | 0.00 | -0.01 | 0.00 |
| 15-19 | 0.00 | 0.00 | 0.00 | 0.00 | 0.14 | 0.00 | 0.00 | 0.00 | -0.01 | 0.00 | 0.01 | 0.02 | -0.04 | 0.02 | 0.13 |
| 20-24 | 0.00 | 0.00 | 0.05 | 0.00 | 0.25 | 0.00 | 0.00 | 0.00 | 0.00 | 0.00 | 0.01 | 0.02 | -0.10 | 0.07 | 0.30 |
| 25-29 | 0.00 | 0.00 | 0.02 | 0.01 | 0.24 | 0.00 | 0.02 | 0.00 | -0.01 | 0.00 | 0.00 | -0.03 | -0.04 | 0.05 | 0.26 |
| 30-34 | 0.01 | 0.02 | 0.02 | 0.01 | 0.17 | 0.01 | 0.01 | 0.00 | 0.00 | 0.00 | 0.00 | 0.00 | 0.02 | 0.03 | 0.28 |
| 35-39 | 0.01 | 0.00 | 0.09 | 0.04 | 0.06 | 0.00 | 0.00 | 0.00 | 0.00 | 0.00 | 0.02 | -0.03 | 0.05 | 0.06 | 0.29 |
| 40-44 | 0.01 | 0.00 | 0.10 | 0.05 | 0.03 | 0.00 | 0.01 | -0.01 | 0.08 | 0.00 | 0.01 | -0.04 | 0.08 | 0.10 | 0.44 |
| 45-49 | 0.01 | 0.02 | 0.13 | 0.02 | 0.06 | 0.02 | 0.01 | 0.02 | 0.10 | 0.00 | 0.02 | -0.04 | 0.06 | 0.16 | 0.59 |
| 50-54 | 0.03 | 0.00 | 0.18 | 0.03 | 0.01 | 0.01 | 0.01 | 0.01 | 0.16 | 0.00 | 0.01 | -0.03 | 0.10 | 0.15 | 0.66 |
| 55-59 | 0.05 | 0.02 | 0.15 | 0.02 | 0.03 | 0.03 | 0.01 | 0.00 | 0.34 | 0.00 | 0.00 | -0.02 | 0.05 | 0.14 | 0.83 |
| 60-64 | 0.05 | 0.05 | 0.16 | 0.00 | 0.02 | 0.00 | 0.01 | 0.00 | 0.23 | 0.00 | 0.02 | -0.02 | 0.02 | 0.18 | 0.72 |
| 65-69 | 0.06 | 0.01 | 0.17 | 0.01 | 0.02 | 0.00 | 0.00 | 0.01 | 0.26 | 0.00 | 0.01 | 0.00 | 0.03 | 0.15 | 0.72 |
| 70-74 | 0.04 | 0.03 | 0.14 | 0.00 | 0.00 | 0.00 | 0.00 | 0.00 | 0.17 | 0.00 | 0.03 | -0.01 | 0.01 | 0.14 | 0.54 |
| 75-79 | 0.08 | 0.03 | 0.06 | 0.00 | 0.00 | 0.03 | 0.00 | -0.01 | 0.07 | 0.00 | 0.01 | 0.00 | 0.00 | 0.15 | 0.41 |
| 80-84 | 0.04 | 0.01 | 0.00 | 0.00 | 0.00 | 0.00 | -0.01 | 0.00 | 0.04 | 0.00 | 0.01 | 0.00 | -0.01 | 0.07 | 0.16 |
| 85 + | 0.00 | -0.01 | -0.14 | 0.00 | 0.00 | 0.00 | 0.01 | 0.00 | -0.01 | 0.00 | -0.02 | 0.00 | 0.00 | 0.01 | -0.16 |
| **Totals** | **0.38** | **0.17** | **1.15** | **0.18** | **1.10** | **0.09** | **0.07** | **0.04** | **1.43** | **0.41** | **0.15** | **-0.17** | **0.29** | **1.66** | **6.96** |

Note: Table entries represent the contribution, in years, to the total difference in life expectancy. Some of these values may be negative.
