# Supplementary material for: Contributors to Wisconsin’s persistent black-white gap in life expectancy
Source: BMC Public Health. 2019 Jul 5;19:891. doi: 10.1186/s12889-019-7145-y (PMC6612087; doi:10.1186/s12889-019-7145-y)
Supplement: Supplementary file 3 — Age and cause decomposition of the 7.34-year difference in life expectancy between non-Hispanic black and non-Hispanic white males in Wisconsin, 2014–16. This table shows the contribution, in years, of each age group and cause of death to the total difference in life expectancy between non-Hispanic black and non-Hispanic white males in Wisconsin from 2014–16. (PDF 95 kb) [file 12889_2019_7145_MOESM3_ESM.pdf]

Additional file 3. Age and cause decomposition of the 7.34-year difference in life expectancy between non-Hispanic black and non-Hispanic white males in Wisconsin, 2014-16

Age groups	Cerebrovascular disease	Diabetes	Heart disease	HIV	Homicide	Hypertension	Influenza & pneumonia	Liver disease	Malignant neoplasms	Perinatal conditions	Respiratory disease	Suicide	Unintentional injuries	All other causes	Total
0	0.00	0.00	0.00	0.00	0.01	0.00	0.00	0.00	0.00	0.36	0.00	0.00	0.06	0.25	0.69
1-4	0.00	0.00	0.00	0.00	-0.01	0.00	0.00	0.00	-0.01	0.01	0.00	0.00	0.03	0.02	0.05
5-9	0.00	0.00	0.00	0.00	0.00	0.00	0.00	0.00	0.01	0.00	0.00	0.00	0.03	0.03	0.08
10-14	0.00	0.00	0.00	0.00	0.02	0.00	0.00	0.00	-0.01	0.00	0.00	0.02	0.03	0.03	0.08
15-19	0.00	0.00	0.00	0.00	0.16	0.00	0.00	0.00	0.00	0.00	0.00	-0.02	0.06	0.01	0.21
20-24	0.00	0.00	0.02	0.02	0.37	0.00	0.00	0.00	0.01	0.00	0.01	-0.02	-0.04	0.01	0.37
25-29	0.00	0.00	0.04	0.00	0.33	0.00	0.01	0.00	-0.01	0.00	0.00	-0.05	-0.07	0.02	0.28
30-34	0.00	0.00	0.05	0.01	0.27	0.00	0.00	-0.01	0.01	0.00	0.01	-0.05	-0.01	0.05	0.33
35-39	0.00	0.01	0.12	0.00	0.18	0.01	0.01	0.00	-0.01	0.00	0.00	-0.04	0.08	0.09	0.46
40-44	0.04	0.02	0.08	0.01	0.06	0.00	-0.01	-0.01	0.00	0.00	0.01	-0.04	0.03	0.06	0.25
45-49	0.01	0.05	0.12	0.01	0.08	0.02	0.02	0.01	0.07	0.00	0.01	-0.05	0.12	0.08	0.55
50-54	0.03	0.02	0.16	0.02	0.03	0.03	0.00	0.03	0.18	0.00	0.01	-0.04	0.09	0.09	0.66
55-59	0.05	0.02	0.28	0.02	0.02	0.01	0.01	0.05	0.31	0.00	0.00	-0.02	0.11	0.17	1.02
60-64	0.04	0.03	0.28	0.01	0.01	0.02	0.03	0.03	0.35	0.00	0.01	-0.01	0.09	0.12	1.02
65-69	0.02	0.04	0.19	0.01	0.01	0.00	0.00	-0.01	0.27	0.00	0.02	-0.01	0.05	0.19	0.79
70-74	0.04	0.04	0.22	0.00	0.01	0.01	0.01	0.00	0.19	0.00	0.00	-0.01	0.01	0.12	0.63
75-79	0.02	0.03	0.05	0.00	0.00	0.02	0.00	0.00	0.00	0.00	0.01	0.00	-0.03	0.03	0.12
80-84	0.01	0.00	0.03	0.00	0.00	0.01	0.00	0.00	0.09	0.00	-0.01	0.00	0.00	0.01	0.12
85 +	-0.02	0.01	-0.19	0.00	0.00	0.02	-0.03	0.00	-0.05	0.00	-0.01	0.00	-0.02	-0.06	-0.35
**Totals**	**0.24**	**0.26**	**1.43**	**0.11**	**1.56**	**0.14**	**0.04**	**0.08**	**1.42**	**0.37**	**0.09**	**-0.36**	**0.63**	**1.33**	**7.34**

Note: Table entries represent the contribution, in years, to the total difference in life expectancy. Some of these values may be negative.
