# Supplementary material for: Contributors to Wisconsin’s persistent black-white gap in life expectancy
Source: BMC Public Health. 2019 Jul 5;19:891. doi: 10.1186/s12889-019-7145-y (PMC6612087; doi:10.1186/s12889-019-7145-y)
Supplement: Supplementary file 4 — Age and cause decomposition of the 5.73-year difference in life expectancy between non-Hispanic black and non-Hispanic white females in Wisconsin, 1999–01. This table shows the contribution, in years, of each age group and cause of death to the total difference in life expectancy between non-Hispanic black and non-Hispanic white females in Wisconsin from 1999–01. (PDF 93 kb) [file 12889_2019_7145_MOESM4_ESM.pdf]

Additional file 4. Age and cause decomposition of the 5.73-year difference in life expectancy between non-Hispanic black and non-Hispanic white females in Wisconsin, 1999-01

Age groups	Cerebrovascular disease	Diabetes	Heart disease	HIV	Homicide	Hypertension	Influenza & pneumonia	Liver disease	Malignant neoplasms	Perinatal conditions	Respiratory disease	Suicide	Unintentional injuries	All other causes	Total
0	0.00	0.00	-0.01	0.00	0.02	0.00	0.04	0.00	0.00	0.35	0.01	0.00	0.03	0.27	0.71
1-4	0.00	0.00	0.01	0.00	0.05	0.00	0.00	0.00	0.00	0.01	0.02	0.00	0.03	0.02	0.13
5-9	0.00	0.00	0.00	0.01	0.01	0.00	0.00	0.00	0.00	0.00	0.00	0.00	0.01	0.01	0.03
10-14	0.00	0.00	0.00	0.00	0.02	0.00	0.00	0.00	0.00	0.00	0.00	-0.01	0.00	0.04	0.06
15-19	0.00	0.01	0.01	0.00	0.02	0.00	0.00	0.00	0.00	0.00	0.00	0.00	-0.04	0.00	0.00
20-24	0.00	0.01	0.02	0.01	0.05	0.00	0.02	0.00	-0.01	0.00	0.01	0.00	0.01	0.07	0.17
25-29	0.01	0.01	0.02	0.00	0.03	0.00	0.01	0.00	0.02	0.00	0.01	-0.01	-0.02	0.07	0.14
30-34	0.00	0.02	0.01	0.02	0.02	0.00	0.00	0.00	-0.01	0.00	0.01	-0.01	0.01	0.05	0.12
35-39	0.02	0.00	0.10	0.02	0.02	0.00	0.01	-0.01	0.05	0.00	0.01	0.01	0.02	0.12	0.37
40-44	0.03	0.01	0.05	0.02	0.02	0.00	0.00	0.00	0.09	0.00	0.02	0.00	0.01	0.10	0.35
45-49	0.01	0.01	0.14	0.03	0.00	0.01	0.01	0.01	0.10	0.00	0.01	0.01	-0.01	0.11	0.44
50-54	0.04	0.05	0.18	0.01	0.01	0.01	0.00	0.00	0.06	0.00	0.01	-0.01	0.01	0.15	0.52
55-59	0.05	0.07	0.18	0.00	0.01	0.01	-0.01	-0.01	0.05	0.00	0.01	-0.01	-0.01	0.09	0.42
60-64	0.07	0.07	0.25	0.00	0.01	0.01	0.00	0.00	0.07	0.00	-0.01	-0.01	0.01	0.12	0.59
65-69	0.02	0.08	0.22	0.00	0.01	0.03	0.01	-0.01	0.18	0.00	-0.01	0.00	-0.01	0.10	0.59
70-74	0.06	0.13	0.17	0.00	0.00	0.00	0.00	-0.01	0.05	0.00	0.00	0.00	-0.01	0.19	0.58
75-79	0.07	0.04	0.17	0.00	0.01	0.03	0.04	-0.01	0.06	0.00	-0.01	0.00	0.00	0.11	0.51
80-84	0.05	0.01	0.01	0.00	0.00	0.00	-0.01	0.00	0.02	0.00	-0.01	0.00	0.00	0.08	0.16
85 +	0.00	0.07	-0.19	0.00	0.01	0.04	-0.05	0.00	0.00	0.00	-0.03	0.00	-0.06	0.05	-0.16
**Totals**	**0.43**	**0.58**	**1.32**	**0.11**	**0.30**	**0.13**	**0.07**	**-0.05**	**0.74**	**0.36**	**0.05**	**-0.04**	**-0.02**	**1.75**	**5.73**

Note: Table entries represent the contribution, in years, to the total difference in life expectancy. Some of these values may be negative.
