# Supplementary material for: Contributors to Wisconsin’s persistent black-white gap in life expectancy
Source: BMC Public Health. 2019 Jul 5;19:891. doi: 10.1186/s12889-019-7145-y (PMC6612087; doi:10.1186/s12889-019-7145-y)
Supplement: Supplementary file 5 — Age and cause decomposition of the 5.82-year difference in life expectancy between non-Hispanic black and non-Hispanic white females in Wisconsin, 2007–09. This table shows the contribution, in years, of each age group and cause of death to the total difference in life expectancy between non-Hispanic black and non-Hispanic white females in Wisconsin from 2007–09. (PDF 95 kb) [file 12889_2019_7145_MOESM5_ESM.pdf]

Additional file 5. Age and cause decomposition of the 5.82-year difference in life expectancy between non-Hispanic black and non-Hispanic white females in Wisconsin, 2007-09

Age groups	Cerebrovascular disease	Diabetes	Heart disease	HIV	Homicide	Hypertension	Influenza & pneumonia	Liver disease	Malignant neoplasms	Perinatal conditions	Respiratory disease	Suicide	Unintentional injuries	All other causes	Total
0	0.00	0.00	0.00	0.00	0.01	0.00	0.00	0.00	0.00	0.24	0.01	0.00	0.03	0.26	0.54
1-4	0.00	0.00	0.01	0.00	0.01	0.00	0.00	0.00	-0.01	0.01	0.00	0.00	0.00	0.03	0.05
5-9	0.01	0.00	0.00	0.00	0.00	0.00	0.00	0.00	-0.01	0.01	0.00	0.00	0.02	0.01	0.03
10-14	0.00	0.00	0.01	0.00	0.00	0.00	0.01	0.00	0.00	0.00	0.01	0.00	0.02	0.00	0.04
15-19	0.00	0.00	0.00	0.00	0.02	0.00	0.00	0.00	0.01	0.00	0.01	0.00	-0.02	0.02	0.03
20-24	0.01	0.00	0.00	0.00	0.05	0.00	0.00	0.00	-0.01	0.00	0.00	-0.01	-0.03	0.03	0.03
25-29	0.01	0.00	0.02	0.01	0.06	0.00	0.01	0.01	0.00	0.00	0.01	0.02	-0.01	0.03	0.17
30-34	0.02	0.00	0.01	0.01	0.00	0.00	0.00	0.00	-0.02	0.00	0.01	0.00	0.02	0.08	0.11
35-39	0.01	0.01	0.06	0.02	0.01	0.00	0.00	0.00	0.02	0.00	0.00	-0.02	0.03	0.09	0.21
40-44	0.01	0.01	0.09	0.00	0.02	0.01	0.01	0.01	0.04	0.00	0.02	-0.01	0.05	0.13	0.38
45-49	0.04	0.02	0.18	0.01	0.00	0.01	0.01	0.03	0.09	0.00	0.02	-0.01	0.05	0.14	0.60
50-54	0.05	0.04	0.17	0.02	0.00	0.00	0.00	0.01	0.21	0.00	0.02	-0.01	0.03	0.15	0.70
55-59	0.05	0.04	0.24	0.01	0.00	0.01	0.02	0.00	0.19	0.00	0.01	-0.01	0.02	0.17	0.76
60-64	0.05	0.04	0.23	0.00	0.00	0.03	0.01	0.01	0.16	0.00	-0.02	-0.01	0.00	0.18	0.66
65-69	0.05	0.04	0.12	0.01	0.01	0.02	0.00	-0.01	0.04	0.00	0.02	0.00	0.01	0.14	0.42
70-74	0.06	0.03	0.18	0.00	0.00	0.00	0.00	0.01	0.14	0.00	-0.06	0.00	0.01	0.08	0.46
75-79	0.00	0.06	0.09	0.00	0.00	0.02	0.00	-0.01	0.08	0.00	-0.02	0.00	-0.02	0.15	0.35
80-84	0.06	0.04	0.12	0.00	0.00	0.02	-0.01	0.00	0.04	0.00	-0.01	0.00	-0.01	0.10	0.35
85 +	0.00	0.06	-0.04	0.00	0.00	0.01	-0.02	0.00	-0.01	0.00	-0.06	0.00	-0.04	0.03	-0.08
**Totals**	**0.42**	**0.38**	**1.48**	**0.09**	**0.18**	**0.13**	**0.04**	**0.04**	**0.94**	**0.25**	**-0.06**	**-0.07**	**0.17**	**1.83**	**5.82**

Note: Table entries represent the contribution, in years, to the total difference in life expectancy. Some of these values may be negative.
