# Supplementary material for: Contributors to Wisconsin’s persistent black-white gap in life expectancy
Source: BMC Public Health. 2019 Jul 5;19:891. doi: 10.1186/s12889-019-7145-y (PMC6612087; doi:10.1186/s12889-019-7145-y)
Supplement: Supplementary file 6 — Age and cause decomposition of the 5.61-year difference in life expectancy between non-Hispanic black and non-Hispanic white females in Wisconsin, 2014–16. This table shows the contribution, in years, of each age group and cause of death to the total difference in life expectancy between non-Hispanic black and non-Hispanic white females in Wisconsin from 2014–16. (PDF 93 kb) [file 12889_2019_7145_MOESM6_ESM.pdf]

Additional file 6. Age and cause decomposition of the 5.61-year difference in life expectancy between non-Hispanic black and non-Hispanic white females in Wisconsin, 2014-16

Age groups	Cerebrovascular disease	Diabetes	Heart disease	HIV	Homicide	Hypertension	Influenza & pneumonia	Liver disease	Malignant neoplasms	Perinatal conditions	Respiratory disease	Suicide	Unintentional injuries	All other causes	Total
0	0.01	0.00	0.01	0.00	0.02	0.00	0.00	0.00	0.00	0.38	0.00	0.00	0.02	0.21	0.66
1-4	0.01	0.00	0.01	0.00	0.02	0.00	-0.01	0.00	0.02	0.00	0.00	0.00	0.01	0.06	0.11
5-9	0.00	0.00	0.01	0.00	0.00	0.00	0.01	0.00	0.00	0.00	0.01	0.00	0.00	-0.01	0.02
10-14	0.00	0.00	0.00	0.00	0.01	0.00	0.01	0.00	-0.01	0.00	0.01	0.00	0.00	0.03	0.06
15-19	0.00	0.00	0.00	0.00	0.06	0.00	0.00	0.00	0.00	0.00	0.01	0.00	0.00	0.02	0.08
20-24	0.01	0.01	0.01	0.00	0.04	0.00	0.00	0.00	-0.01	0.00	0.00	0.00	0.01	0.03	0.11
25-29	0.01	0.00	0.01	0.00	0.03	0.00	0.00	0.00	-0.01	0.00	0.00	-0.01	0.03	0.04	0.10
30-34	0.00	0.00	0.05	0.00	0.02	0.00	0.00	-0.01	0.03	0.00	0.00	-0.02	-0.01	0.05	0.11
35-39	0.01	0.01	0.05	0.01	0.01	0.00	0.00	0.00	0.03	0.00	0.00	0.00	-0.01	0.09	0.19
40-44	0.02	0.02	0.08	0.01	0.01	0.00	0.01	0.00	0.07	0.00	0.00	-0.02	-0.01	0.09	0.27
45-49	0.04	0.02	0.13	0.01	0.01	0.01	0.00	0.02	0.04	0.00	0.01	-0.01	0.08	0.16	0.53
50-54	0.06	0.01	0.21	0.02	0.01	0.02	0.01	0.02	0.13	0.00	0.03	-0.02	0.06	0.10	0.65
55-59	0.07	0.03	0.17	0.00	0.00	0.01	0.01	0.02	0.16	0.00	0.05	-0.01	0.02	0.16	0.67
60-64	0.05	0.06	0.17	0.00	0.00	0.01	0.01	0.01	0.15	0.00	0.05	-0.01	0.03	0.20	0.73
65-69	0.04	0.05	0.18	0.00	0.00	0.04	0.01	-0.01	0.23	0.00	0.00	0.00	0.02	0.15	0.69
70-74	0.10	0.07	0.13	0.00	0.00	0.03	0.00	0.00	0.16	0.00	0.02	0.00	-0.01	0.15	0.64
75-79	0.03	0.05	0.11	0.00	0.00	0.02	0.01	0.00	0.01	0.00	-0.01	0.00	0.00	0.17	0.38
80-84	0.03	0.04	0.06	0.00	0.00	0.00	-0.01	0.00	0.01	0.00	-0.03	0.00	-0.01	0.04	0.13
85 +	-0.07	0.04	-0.27	0.00	0.00	0.00	-0.05	0.00	-0.01	0.00	-0.05	0.00	-0.10	-0.03	-0.53
**Totals**	**0.40**	**0.41**	**1.12**	**0.05**	**0.24**	**0.13**	**0.02**	**0.05**	**1.00**	**0.38**	**0.08**	**-0.11**	**0.13**	**1.71**	**5.61**

Note: Table entries represent the contribution, in years, to the total difference in life expectancy. Some of these values may be negative.
