# Supplementary material for: Contributors to Wisconsin’s persistent black-white gap in life expectancy
Source: BMC Public Health. 2019 Jul 5;19:891. doi: 10.1186/s12889-019-7145-y (PMC6612087; doi:10.1186/s12889-019-7145-y)
Supplement: Supplementary file 10 — Age and cause decomposition of the 2.44-year difference in life expectancy between non-Hispanic black females in Wisconsin and non-Hispanic black females in the U.S., 2014–16. This table shows the contribution, in years, of each age group and cause of death to the total difference in life expectancy between non-Hispanic black females in Wisconsin and non-Hispanic black females in the U.S. from 2014–16. (PDF 95 kb) [file 12889_2019_7145_MOESM10_ESM.pdf]

Additional file 10. Age and cause decomposition of the 2.44-year difference in life expectancy between non-Hispanic black females in Wisconsin and non-Hispanic black females in the U.S., 2014-16

Age groups	Cerebrovascular disease	Diabetes	Heart disease	HIV	Homicide	Hypertension	Influenza & pneumonia	Liver disease	Malignant neoplasms	Perinatal conditions	Respiratory disease	Suicide	Unintentional injuries	All other causes	Total
0	0.01	0.00	0.00	0.00	0.01	0.00	0.00	0.00	0.00	0.14	0.00	0.00	0.00	0.10	0.27
1-4	0.01	0.00	0.00	0.00	0.01	0.00	0.00	0.00	0.01	0.00	0.00	0.00	-0.01	0.05	0.06
5-9	0.00	0.00	0.00	0.00	0.00	0.00	0.01	0.00	0.00	0.00	0.00	0.00	-0.01	-0.01	-0.01
10-14	0.00	0.00	0.00	0.00	0.01	0.00	0.01	0.00	-0.01	0.00	0.00	0.00	0.00	0.02	0.03
15-19	0.00	0.00	-0.01	0.00	0.04	0.00	0.00	0.00	0.00	0.00	0.00	0.01	0.01	0.00	0.06
20-24	0.00	0.01	0.00	0.00	0.01	0.00	0.00	0.00	-0.01	0.00	0.00	0.01	0.03	0.00	0.05
25-29	0.01	-0.01	0.00	-0.01	0.01	0.00	0.00	0.00	-0.01	0.00	0.00	0.01	0.05	0.01	0.06
30-34	-0.01	-0.01	0.02	-0.01	0.01	0.00	0.00	0.00	0.02	0.00	0.00	0.00	0.01	0.00	0.00
35-39	0.00	0.00	0.01	-0.01	0.00	0.00	0.00	0.00	0.00	0.00	-0.01	0.00	0.00	0.02	0.01
40-44	0.01	0.01	0.01	-0.01	0.00	-0.01	0.01	0.00	0.03	0.00	-0.01	-0.01	0.00	0.02	0.06
45-49	0.02	0.01	0.05	-0.01	0.01	0.00	0.00	0.02	-0.01	0.00	0.00	0.00	0.07	0.07	0.23
50-54	0.03	-0.02	0.08	0.00	0.00	0.00	0.00	0.02	0.04	0.00	0.02	0.00	0.05	0.01	0.25
55-59	0.04	-0.01	0.03	-0.01	0.00	0.00	0.00	0.02	0.04	0.00	0.03	0.00	0.02	0.04	0.19
60-64	0.00	0.02	0.01	-0.01	0.00	-0.01	0.00	0.01	0.03	0.00	0.05	0.00	0.03	0.08	0.20
65-69	0.00	0.01	0.03	-0.01	0.00	0.02	0.00	-0.01	0.14	0.00	0.02	0.00	0.02	0.05	0.27
70-74	0.07	0.03	0.00	0.00	0.00	0.02	-0.01	0.01	0.14	0.00	0.05	0.00	0.00	0.07	0.36
75-79	-0.01	0.02	0.00	0.00	0.00	0.00	0.00	0.00	0.02	0.00	0.03	0.00	0.03	0.11	0.20
80-84	0.00	0.02	0.02	0.00	0.00	-0.02	-0.01	0.01	0.02	0.00	0.00	0.00	0.02	0.04	0.10
85 +	-0.05	0.01	-0.19	0.00	0.00	-0.02	-0.03	0.00	0.03	0.00	0.01	0.00	0.03	0.25	0.05
**Totals**	**0.13**	**0.09**	**0.06**	**-0.08**	**0.10**	**-0.02**	**-0.02**	**0.07**	**0.50**	**0.14**	**0.21**	**0.02**	**0.33**	**0.93**	**2.44**

Note: Table entries represent the contribution, in years, to the total difference in life expectancy. Some of these values may be negative. To avoid double counting non-Hispanic blacks in Wisconsin, we removed them from U.S. data in these estimates.
